# Epithelioid Trophoblastic Tumour: A Case with Genetic Linkage to a Child Born over Seventeen Years Prior, Successfully Treated with Surgery and Pembrolizumab

**DOI:** 10.3390/curroncol28060446

**Published:** 2021-12-13

**Authors:** David Pisani, Jean Calleja-Agius, Riccardo Di Fiore, John J. O’Leary, James P. Beirne, Sharon A. O’Toole, Ana Felix, Ian Said-Huntingford

**Affiliations:** 1 Department of Histopathology, Mater Dei Hospital, MSD2090 Msida, Malta; david.c.pisani@gov.mt (D.P.); ian.said-huntingford@gov.mt (I.S.-H.); 2 Department of Anatomy, Faculty of Medicine and Surgery, University of Malta, MSD2080 Msida, Malta; riccardo.difiore@um.edu.mt; 3 Sbarro Institute for Cancer Research and Molecular Medicine, Center for Biotechnology, College of Science and Technology, Temple University, Philadelphia, PA 19122, USA; 4 Department of Histopathology, Trinity College Dublin, Trinity St. James’s Cancer Institute, St. James Hospital, D08 NHY1 Dublin, Ireland; olearyjj@tcd.ie; 5 Department of Gynaecological Oncology, Trinity St James’s Cancer Institute, St. James Hospital, D08 NHY1 Dublin, Ireland; JBeirne@stjames.ie; 6 Departments of Obstetrics and Gynaecology and Histopathology, Trinity St James’s Cancer Institute, Trinity College Dublin, D08 NHY1 Dublin, Ireland; SHOTOOLE@tcd.ie; 7 Department of Pathology, Campo dos Mártires da Pátria, Instituto Portugues de Oncologia de Lisboa, NOVA Medical School, UNL, 130, 1169-056 Lisbon, Portugal; ana.felix@nms.unl.pt

**Keywords:** epithelioid trophoblastic tumour, gestational trophoblastic tumour, rare gynaecological tumour

## Abstract

Epithelioid trophoblastic tumours are rare neoplasms showing differentiation towards the chorion leave-type intermediate cytotrophoblast, with only a handful of cases being reported in the literature. These tumours are slow-growing and are typically confined to the uterus for extended periods of time. While the pathogenesis is unclear, they are thought to arise from a remnant intermediate trophoblast originating from prior normal pregnancies or, less frequently, gestational trophoblastic tumours. A protracted time period between the gestational event and tumour development is typical. This case describes a 49-year-old previously healthy female who presented with a completely asymptomatic uterine mass, discovered incidentally during a routine gynaecological assessment. The pathological analysis of the hysterectomy specimen confirmed an epithelioid trophoblastic tumour, involving the uterus and cervix. This is a rare gynaecological tumour. A comparative short tandem repeat analysis revealed genetic similarities to a previous healthy gestation seventeen years prior. She was successful treated with adjuvant pembrolizumab, with no evidence of disease recurrence to date.

## 1. Introduction

Epithelioid trophoblastic tumours (ETT) are distinct, rare neoplasms arising from a chorionic-type intermediate trophoblast. We describe a case of a middle-aged female who was diagnosed with an ETT following the incidental detection of a uterine mass during a routine gynaecological assessment. Tumour genetic analysis revealed a link with a healthy gestation seventeen years prior.

## 2. Case Presentation

A middle-aged female was referred from a private clinic in May of 2020 for investigation of a suspicious uterine mass incidentally detected on ultrasonographic studies. The patient was a 49-year-old female who was being followed up routinely by her private gynaecologist. She was completely asymptomatic and denied menorrhagia, post-coital bleeding, or vaginal discharge. She had suffered from heavy menstrual bleeding in 2012 and had been successfully treated with a levonorgestrel-releasing intrauterine system. She was a mother to two healthy children, with a female child born in 2003 and a male child born in 2006. Both children were born by normal vaginal delivery and no obstetric complications were reported. There was no history of miscarriages. She had no significant medical or surgical history of note and was not on any regular medications. Gynaecological examination revealed a bulky uterus, comparable to the size of a fourteen-week gestation.

**Figure 1:**
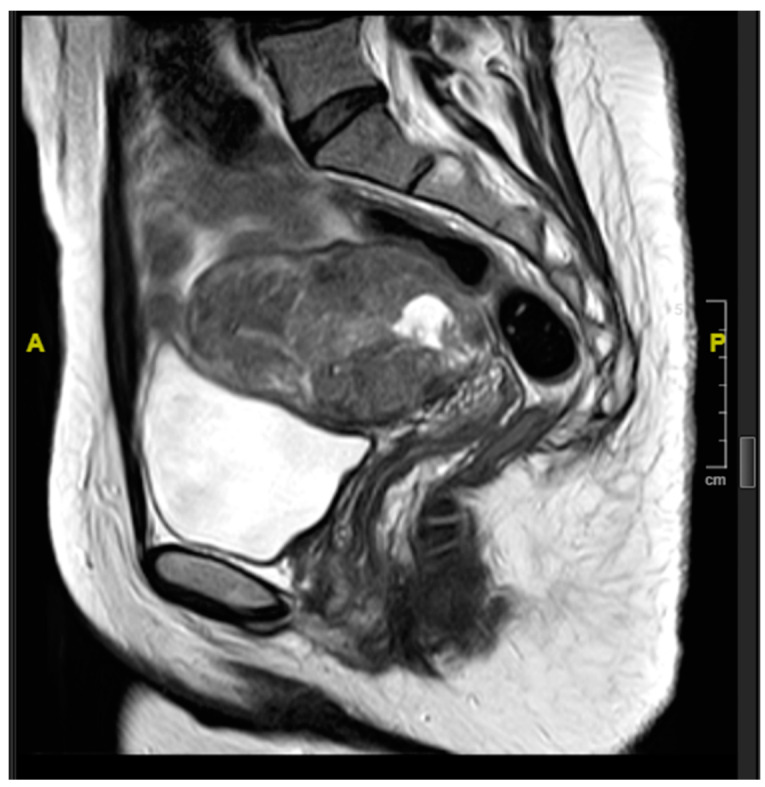
Sagittal T2-weighted MRI showing mass centred on the lower uterine segment with associated uterine distortion.

**Figure 2:**
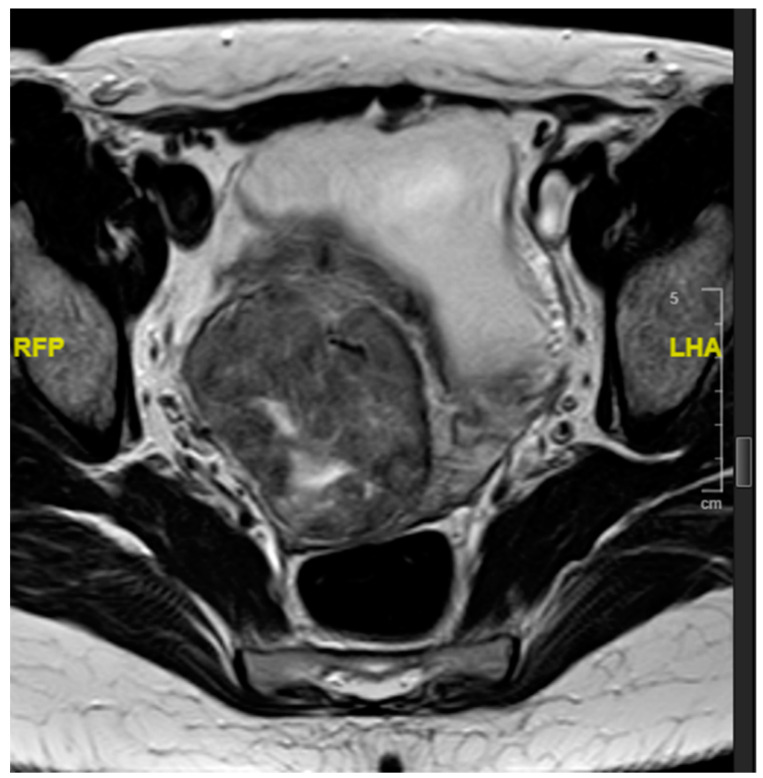
Transverse T2-weighted MRI surrounding the lower uterine cavity and distorting the uterine canal.

Blood investigations, including a complete blood count, a renal profile and liver function tests, were within normal limits. Serum levels of carcinoembryonic antigen (CEA), cancer-antigen 125 (CA 125), and cancer antigen 19.9 (CA 19.9) were within normal limits and β-human chorionic gonadotrophin (βhCG) levels were normal, at 1.4 mIU/L (local reference range 0–2.7 mIU/L). Computed tomographic assessment showed a 7 cm mass in the uterus, centred on the cervico-uterine junction. Magnetic resonance imaging showed an approximately 5 × 6 cm^2^ soft tissue circumferential mass arising from the lower uterus, showing an intermediate T1 and T2 signal. The mass had a 1.5 cm cystic component centrally. The tumour distorted the uterine cavity, but did not extend into the cervix or para-uterine space. No enlarged pelvic lymph nodes were identified (Figure 1 and Figure 2). The mass showed heterogenous enhancement and demonstrated central necrosis. No associated pelvic or abdominal lymphadenopathy was noted and there was no evidence of distant metastatic spread.

In view of the strong radiological suspicion of malignancy, following discussion with the patient, a routine total abdominal hysterectomy and bilateral salpingo-oöphorectomy were carried out. The surgical assessment of the abdomen and pelvis showed no gross evidence of metastatic disease. A biopsy of the greater omentum was also submitted for pathological analysis.

The pathological analysis of the uterus showed the presence of a mass, centred on the posterior aspect of the lower uterine segment, measuring 55 mm in its greatest dimension. The mass had a tan-brown, focally haemorrhagic cut surface and showed foci suspicious for necrosis. The tumour was relatively well-circumscribed, but extended into the posterior cervical lip. The tumour was associated with minimal distortion of the uterine cavity. The rest of the uterus and both adnexae were grossly unremarkable.

**Figure 3:**
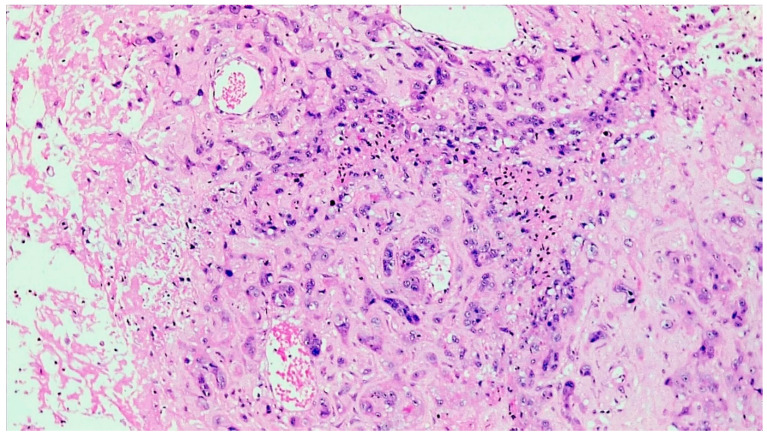
Histological analysis showed a tumour comprised of neoplastic trophoblast cells with abundant eosinophilic cytoplasm and indistinct cell borders, infiltrating and replacing vessel walls (H&E, ×200).

**Figure 4:**
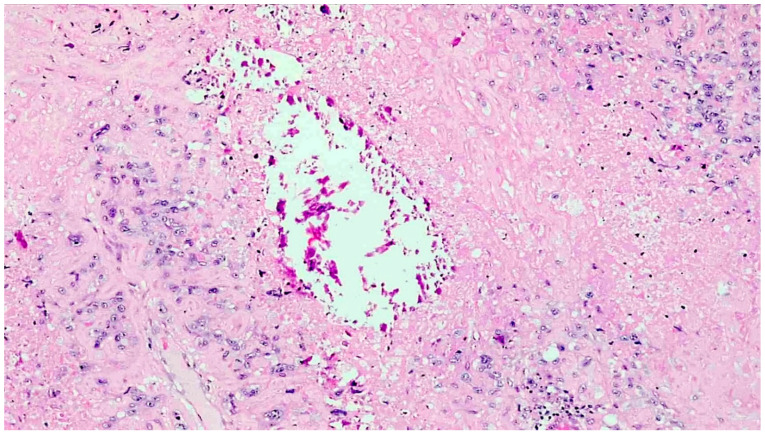
The neoplastic cells were embedded in abundant eosinophilic extracellular material, bearing a morphological semblance to keratin, and dystrophic calcifications were noted throughout the tumour (H&E, ×200).

The histopathological analysis showed a tumour comprised of atypical mononucleate epithelioid cells with abundant eosinophilic cytoplasm and ill-defined cell membranes, organised in infiltrative nests and sheets (Figure 3). Scattered, interspersed, multinucleated, pleomorphic tumour cells were also present. Extensive geographic necrosis was appreciated. Multifocally, the neoplastic cells were embedded in eosinophilic, hyaline material reminiscent of extracellular keratin. The neoplastic cells extended around the smaller myometrial blood vessels, with extensive associated deposition of fibrin. Dystrophic calcific deposits were also noted throughout the tumour (Figure 4). The tumour also encircled the endometrial and endocervical glands, completely replacing the mucosa in areas.

**Figure 5:**
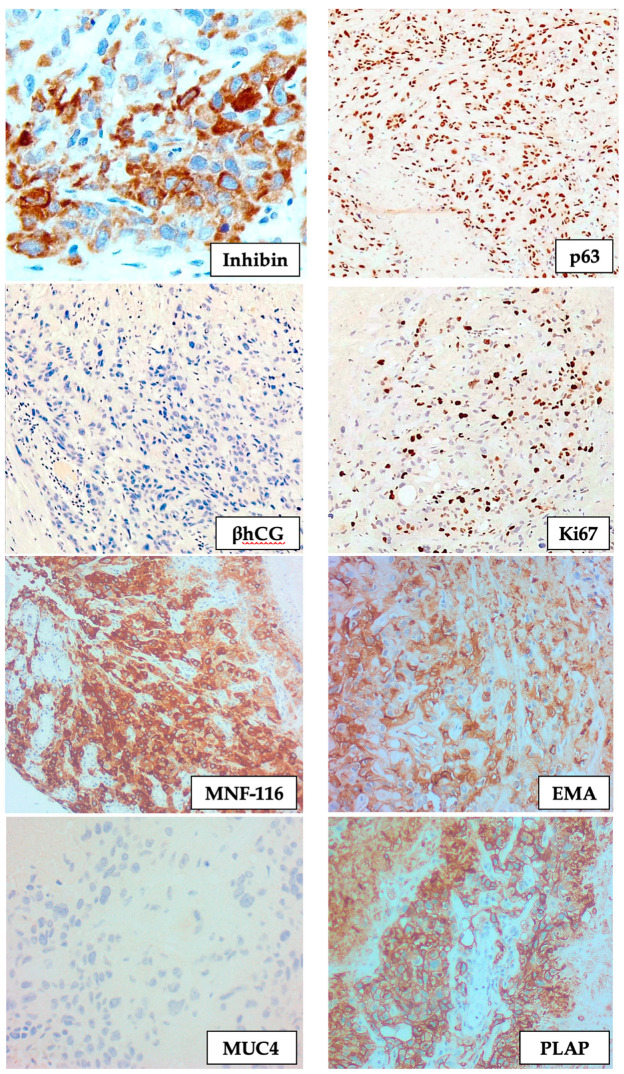
Immunohistochemistry showing the expression of inhibin, p63, MNF-116, EMA, and PLAP, together with absent βhCG and MUC4 staining, and a low Ki67 proliferation fraction (IHC, ×200).

The immunohistochemical studies showed a strong and diffuse expression of cytokeratin stains (MNF116 and AE1/AE3), together with placental alkaline phosphatase (PLAP), inhibin, epithelial membrane antigen (EMA), and E-cadherin. The differentiation towards a chorion leave-type intermediate trophoblastic lineage was further confirmed with strong expression p63, very focal expression of βhCG, and absent mucin-4 (MUC4) staining. A Ki67 proliferation fraction of 20% was demonstrated immunohistochemically (Figure 5). The findings were those of an epithelioid trophoblastic tumour.

The tumour infiltrated the outer half of the myometrium together with the upper cervix. However, the tumour did not reach the serosa or the uterine parametrium, and the excision was deemed to be complete, with a Fédération Internationale de Gynécologie et d’Obstétrique (FIGO) stage I disease. The omentum was free of metastatic disease.

A comparative short tandem repeat genotypic analysis was performed, comparing the tumour genotype with that obtained from saliva samples taken from the patient, the biological father of both her offspring, and both offspring (Table 1). The presence of non-maternal alleles in the tumour supported the tumour being of gestational origin. Furthermore, genotypic analysis showed that the tumour contained non-maternal alleles that were identical to those found in her first-born child born in 2003, suggesting that this tumour originated from the 2003 gestational event.

Following an uneventful post-operative recovery, as in the case of all local cases of gestational trophoblastic disease, this patient was referred to the Charing Cross Gestational Trophoblastic Disease Service in London, UK. This case was diagnosed during the COVID-19 pandemic (June 2020) and prior to the availability of any COVID-19 vaccinations. At this time, many hospital services were disrupted and there were restrictions on air travel. Since genetic testing linked the ETT to the conception in 2003, the case was deemed to be of high risk and a treatment with pembrolizumab was recommended. This was the preferred treatment option to minimize the risk of neutropaenic episodes. Therefore, the patient was started on pembrolizumab at a dose of 200 mg every three weeks, in accordance with the NHS England Guidelines on the use of Pembrolizumab for Gestational Trophoblastic Neoplasia. She suffered no therapy-related complications and both computed tomographic imaging studies and pelvic magnetic resonance imaging showed no evidence of locoregional recurrence or distant metastatic disease within a twelve-month follow-up period.

## 3. Discussion

Gestational trophoblastic disease constitutes a group of disorders caused by pathological aberrations occurring during placental development. These diseases can be broadly subclassified into three distinct groups: benign trophoblastic lesions (placental-site nodules and exaggerated placental site reactions), hydatidiform moles (partial moles, complete moles, and invasive moles), and true gestational neoplasms (choriocarcinoma, epithelioid trophoblastic tumour, and placental site trophoblastic tumour), with the latter group representing neoplasms recapitulating various stages of placental development [1].

The earliest stage of embryonic trophoblastic differentiation is represented by the trophectoderm, which forms as the outermost layer of the blastocyst. The trophectoderm adjacent to the inner cell mass subsequently differentiates, forming cytotrophoblast together with an early multinuclear primitive syncytium, the latter playing a key role in the early invasion of the maternal vasculature to form blood-filled sinusoids. These sinusoids are subsequently permeated by the cytotrophoblast, forming primary villi, which then develop into secondary villi after the migration of the extraembryonic mesoderm, originating from the embryonic disk [2]. The cytotrophoblast serves as a precursor trophoblast cell, from which other types of trophoblast cells develop. Cytotrophoblastic differentiation into syncytiotrophoblast cells occurs on the surface of the placental villi. Cytotrophoblastic differentiation into intermediate trophoblast is more complex, with three distinct phenotypic forms of intermediate trophoblast being recognised, namely intermediate trophoblast in the trophoblastic columns (which serve as a support column for anchoring villi), implantation-site intermediate trophoblast (serving to anchor the placenta to the uterine wall), and chorionic-type intermediate trophoblast (restricted to the chorionic leave of the foetal membranes) [3]. It is from the latter that ETTs are thought to arise.

ETTs are rare malignancies, with approximately 140 cases described worldwide. These tumours were first classified as a distinct entity by Shih and Kurman in 1998, who described the first cohort of 14 cases. In this series, all tumours were localised to the uterus, cervico-uterine junction, or cervix, and were histologically characterised by sheets and islands of trophoblastic cells, with extensive geographic necrosis, associated extracellular hyaline-like matrix, and scattered calcifications [4], features identical to those identified in the case presented in this paper. With further case reports and series, the disease was found to be the commonest in the 33-to-40 age bracket and, in addition to isolated uterine disease, cases of isolated extrauterine disease (including lung, small bowel, gallbladder, and adnexa) were also described [5].

ETTs bear a strong association with prior gestational events, most frequently normal vaginal delivery together with molar gestations and abortions [6]. However, rare cases have been described without any antecedent pregnancies. The latter cases, however, in all probability represent a cohort of females having suffered a clinically undetected early miscarriage. While the pathogenesis of these tumours is unclear, it appears that they originate from residual, entrapped trophoblastic elements that remain within the uterine wall following a gestational event. An unusual aspect about the case presented in this paper is the genetic linkage with the first gestation, with the occurrence of a completely normal pregnancy three years after and development of disease seventeen years after the first gestation.

Two additional unique features of this case warrant mentioning, in particular the complete absence of clinical symptoms together with normal serum βhCG levels. Most patients present with abnormal vaginal bleeding, amenorrhea, abdominal pain or bloating [5], and often have mild-to-moderate elevations in serum βhCG levels [7]. While the reason for the absence of symptoms in this case is unclear, the exophytic nature of the mass with minimal distortion of the uterine cavity may account for this.

On the histopathological analysis, the differentiation towards chorionic-type intermediate trophoblast is confirmed through the expression of inhibin-α, PLAP, p63, EMA, broad range cytokeratin, and E-cadherin, together with a weak expression of human placental lactogen and βhCG [4]. Given the morphological semblance of this tumour to squamous cell carcinoma, the expression of cytokeratin markers and p63 may further contribute to an erroneous diagnosis and is a well-recognised diagnostic pitfall, particularly given the rarity of this diagnosis.

The behaviour of ETT is difficult to predict; however, within the limits of the data currently available, it appears to be similar to placental site trophoblastic tumours [8]. The metastatic spread at the time of diagnosis is present in approximately one quarter of patients and the recurrence of the disease occurs in roughly one third of patients. Death from disease occurs in one quarter of patients, with the FIGO stage at presentation being the most significant prognostic factor, together with multifocal intrauterine disease and deep myometrial and serosal involvement [7,9,10]. No correlations between the size of the tumour, serum βhCG levels, mitotic activity (or Ki67 proliferation fraction), patient age, or interval between disease and the last gestational event have been established [11,12,13].

No standard treatment protocols are available for the management of ETTs given that large-scale clinical trials are not possible owing to their inherent rarity. ETT, similar to placental site trophoblastic tumour, is a chemoresistant neoplasm. While adjuvant therapy with various combinations of etoposide, cisplatin, actinomycin D, methotrexate, and paclitaxel have been successfully used, a case-by-case approach is mandatory as reports of cases of patients having been successfully treated with radical surgery to remove metastatic or recurrent disease have been documented [5,14,15,16]. Additionally, the expression of PD-L1, PD-L2, B7-H3, VISTA, and CD105 is frequently noted in ETT, suggesting the PD-1/PD-L1 checkpoint inhibition to be a viable therapeutic option for the management of the disease [17]. PD-L1 immunohistochemistry was not performed in this case. This is primarily due to the fact that, since this is a rare tumour, PD-L1 immunohistochemistry is not performed at the local hospital, but at a reference laboratory abroad (Charing Cross Hospital, London, UK), and these particular results were not yet available at the time of writing. In addition, the correlation between PD-L1 immunohistochemistry and the response to immunotherapy has not been validated in the literature and no set standards are available regarding cut off for therapy. Pembrolizumab was used successfully in this case [18,19,20], with no evidence of disease recurrence over a 12-month period.

## 4. Conclusions

In this paper, we describe a case of an epithelioid trophoblastic tumour in a 49-year-old female with genetic linkage to a gestational event seventeen years prior, with an interim unrelated second normal gestation. The patient was successfully treated with surgery and adjuvant pembrolizumab, the latter being a relatively novel agent used in the treatment of these rare neoplasms. The patient was completely asymptomatic and had normal serum βhCG levels, both being unusual for this disease entity. The unusual, albeit distinct, histomorphology and immunophenotype of ETTs are emphasised to avoid diagnostic pathological pitfalls with other neoplastic entities.

## Figures and Tables

**Table 1:** Short tandem repeat analysis across twenty-four loci comparing the tumour with the mother, father, and both children. Loci showing unique links between the tumour and the 2003 gestation are highlighted in blue.

Locus	Chromosome Location	Tumour	Patient (Mother)	Biological Father	Child-2003	Child-2006	Comments
*D3S1358*	3p21.31	15/17	15/15	17/17	15/17	15/17	Non-maternal allele
*vWA*	12p13.31	14/17	17/17	14/16	14/17	16/17	Non-maternal allele (2003)
*D16S539*	16q24.1	11/11	11/11	9/11	11	9/11	Non informative
*CSF1PO*	5q33.3-34	10/11/14	11/14	10/12	10/11	12/14	Non-maternal allele (2003)
*TPOX*	2p23-2per	8/10	8/8	8/10	8/10	8/10	NMA
*Y*	Yq11.221	–	–	2	–	2	Non informative
*Amelogenin*	X:p22.1-22.3 Y:p11.2	XX	XX	XY	XX	XY	-
*D8S1179*	8q24.13	10/13	10/13	13/14	13/13	10/13	Not informative
*D21S11*	21q11.2-q21	29/30/32.2	29/32.2	29/30	30/32.2	29/30	Non-maternal allele
*D18S51*	18q21.33	12/13/17	12/17	12/13	12/13	12/13	Non-maternal allele
*DYS391*	Yq11.21	–	–	10	–	10	-
*D2S441*	2p14	11/11.3/12	11/11.3	12/14.3	11/12	11/14.3	Non-maternal allele (2003)
*D19S433*	19q12	13/14.2	13/13	14/14.2	13/14.2	13/14	Non-maternal allele (2003)
*TH01*	11p15.5	9/9.3/10	9.3/10	9/9	9/10	9/10	Non-maternal allele
*FGA*	4q28	19/22/24	19/24	20/22	22/24	20/24	Non-maternal allele (2003)
*D22S1045*	22q12.3	11/16/18	16/18	11/16	11/16	16/18	Non-maternal allele (2003)
*D5S818*	5q21-31	8/11	11/11	8/11	8/11	8/11	Non-maternal allele
*D13S317*	13q22-31	12/12	12/12	11/12	12/12	12/12	Not informative
*D7S820*	7q11.21-22	11/11	11/11	10/11	11/11	11/11	Not informative
*SE33*	6q14	16/24.2/26.2	16/24.2	26.2/31	24.2/26.2	16/31	Non-maternal allele (2003)
*D10S1248*	10q26.3	12/13	12/13	12/15	12/13	12/15	Not informative
*D1S1656*	1q42.2	11/13/14	13/14	11/19.3	11/13	11/13	Non-maternal allele
*D12S391*	12p13.2	19/20/24	20/24	19/20	19/20	19/20	Non-maternal allele
*D2S1338*	2q35	17/23	17/23	22/23	17/23	22/23	Not informative

## Data Availability

The data presented in this study are available on request from the corresponding author.

## References

[1] Shih I.M. (2007). Gestational trophoblastic neoplasia-pathogenesis and potential therapeutic targets. Lancet Oncol..

[2] Knöfler M., Haider S., Saleh L., Pollheimer J., Gamage T.K., James J. (2019). Human placenta and trophoblast development: Key molecular mechanisms and model systems. Cell Mol. Life Sci..

[3] Cierna Z., Varga I., Danihel L., Kuracinova K., Janegova A. (2016). Intermediate trophoblast—A distinctive, unique and often unrecognized population of trophoblastic cells. Ann. Anat. Anat. Anz..

[4] Shih I.M., Kurman R.J. (1998). Epithelioid trophoblastic tumor: A neoplasm distinct from choriocarcinoma and placental site tropho-blastic tumor simulating carcinoma. Am. J. Surg. Pathol..

[5] Gadducci A., Carinelli S., Guerrieri M.E., Aletti G.D. (2019). Placental site trophoblastic tumor and epithelioid trophoblastic tumor: Clinical and pathological features, prognostic variables and treatment strategy. Gynecol. Oncol..

[6] Horowitz N.S., Goldstein D.P., Berkowitz R.S. (2017). Placental site trophoblastic tumors and epithelioid trophoblastic tumors: Biology, natural history, and treatment modalities. Gynecol. Oncol..

[7] Zhang X., Lü W., Lü B. (2013). Epithelioid Trophoblastic Tumor: An Outcome-Based Literature Review of 78 Reported Cases. Int. J. Gynecol. Cancer.

[8] Seckl M.J., Sebire N.J., Fisher R.A., Golfier F., Massuger L., Sessa C., ESMO Guidelines Working Group (2013). Gestational trophoblastic disease: ESMO Clinical Practice Guidelines for diagnosis, treatment and follow-up. Ann Oncol..

[9] Jiang F., Xiang Y., Guo L.-N. (2018). Laparoscopic diagnosis and treatment of an isolated epithelioid trophoblastic tumor in recto-uterine pouch. J. Obstet. Gynaecol. Res..

[10] Rodríguez-Trujillo A., Martínez-Serrano M.J., Saco A., Torné A. (2017). Two cases of epithelioid trophoblastic tumors in postmenopausal women. Menopause.

[11] Coulson L.E., Kong C.S., Zaloudek C. (2000). Epithelioid trophoblastic tumor of the uterus in a postmenopausal woman: A case report and review of the literature. Am. J. Surg. Pathol..

[12] Fadare O., Parkash V., Carcangiu M.-L., Hui P. (2006). Epithelioid trophoblastic tumor: Clinicopathological features with an emphasis on uterine cervical involvement. Mod. Pathol..

[13] Sung W.J., Shin H.C., Kim M.-K., Kim M.J. (2013). Epithelioid Trophoblastic Tumor: Clinicopathologic and Immunohistochemical Analysis of Three Cases. Korean J. Pathol..

[14] Sobecki-Rausch J., Winder A., Maniar K.P., Hoekstra A.V., Berry E., Novak K., Lurain J.R. (2018). Surgery and Platinum/Etoposide-Based Chemotherapy for the Treatment of Epithelioid Trophoblastic Tumor. Int. J. Gynecol. Cancer.

[15] Lei W., Zhang F., Zheng C., Zhao C., Tu S., Bao Y. (2018). Metastatic epithelioid trophoblastic tumor of the lung: A case report. Medicine.

[16] Kim J.-H., Lee S.K., Hwang S.H., Kim J.-S., Yoon G., Lee Y.-Y., Kim T.-J., Choi C.H., Kim B.-G., Bae D.-S. (2017). Extrauterine epithelioid trophoblastic tumor in hysterectomized woman. Obstet. Gynecol. Sci..

[17] Yang J., Zong L., Wang J., Wan X., Feng F., Xiang Y. (2019). Epithelioid Trophoblastic Tumors: Treatments, Outcomes, and Potential Therapeutic Targets. J. Cancer.

[18] NCCN Guideline Gestational Trophoblastic Neoplasia Version 2.2021; 31 Mar 21. https://www.nccn.org/professionals/physician_gls/pdf/gtn.pdf.

[19] NHS Urgent Clinical Commissioning Policy Statement: Pembrolizumab for Drug-Resistant Gestational Trophoblastic Neo-plasia. NHS England Reference: 170027P. https://www.england.nhs.uk/wp-content/uploads/2018/12/Pembrolizumab-for-drug-resistant-gestational-trophoblastic-neoplasia.pdf.

[20] Eiriksson L., Dean E., Sebastianelli A., Salvador S., Comeau R., Jang J.-H., Bouchard-Fortier G., Osborne R., Sauthier P. (2021). Guideline No. 408: Management of Gestational Trophoblastic Diseases. J. Obstet. Gynaecol. Can..

